# Delivery of Family-Based Treatment for Adolescent Anorexia Nervosa in a Public Health Care Setting: Research Versus Non-Research Specialty Care

**DOI:** 10.3389/fpsyt.2019.01001

**Published:** 2020-01-22

**Authors:** Daniel Le Grange, Sasha Gorrell, Elizabeth K. Hughes, Erin C. Accurso, Michele Yeo, Martin Pradel, Susan M. Sawyer

**Affiliations:** ^1^ Department of Psychiatry, UCSF Weill Institute for Neurosciences, University of California, San Francisco, San Francisco, CA, United States; ^2^ Department of Psychiatry & Behavioral Neuroscience, The University of Chicago, Chicago, IL, United States; ^3^ Department of Paediatrics, The University of Melbourne, Melbourne, VIC, Australia; ^4^ Centre for Adolescent Health, Murdoch Children’s Research Institute, Melbourne, VIC, Australia; ^5^ Department of Adolescent Medicine, Royal Children’s Hospital, Melbourne, VIC, Australia

**Keywords:** anorexia nervosa, adolescents, eating disorder, family-based treatment, treatment outcome

## Abstract

Comparing evidence-based psychotherapy (EBP) to usual care typically demonstrates the superiority of EBPs, although this has not been studied for eating disorders EBPs such as family-based treatment (FBT). The current study set out to examine weight outcomes for adolescents with anorexia nervosa who received FBT through a randomized clinical research trial (RCT, *n* = 54) or non-research specialty care (*n* = 56) at the same specialist pediatric eating disorder service. Weight was recorded throughout outpatient treatment (up to 18 sessions over 6 months), as well as at 6- and 12-month follow-up. Survival curves were used to examine time to weight restoration [greater than 95% median body mass index (mBMI)] as predicted by type of care (RCT vs. non-research specialty care), baseline clinical and demographic characteristics, and their potential interaction. Results did not indicate a significant main effect for type of care, but there was a significant effect for baseline weight (*p* = .03), such that weight restoration was achieved faster across both treatment types for those with a higher initial %mBMI. These data suggest that weight restoration achieved in non-research specialty care FBT was largely similar to that achieved in a controlled research trial.

**Clinical Trial Registration:**

http://www.anzctr.org.au/, identifier ACTRN12610000216011.

## Introduction

Anorexia nervosa (AN) is a particularly pernicious psychiatric illness with significant morbidity and mortality rates (1), considerable distress and impairment (2), and high treatment costs (3). Implementing evidence-based psychotherapy (EBP) for adolescents with AN is a critical endeavor as onset is most commonly in adolescence and early adulthood (4), and early intervention typically yields the most favorable treatment outcomes (5). One potential stumbling block in the implementation of EBPs in usual care is that, while the former outperforms the latter, EBPs may perform more poorly in community clinical settings compared to the research settings in which they were developed and tested (6). For example, strict inclusion criteria, together with potentially different characteristics of patients and families who are willing to participate in a research trial (and subsequently be randomized to either arm of a study) might limit the representativeness of clinical trial data. As such, treatment outcomes from randomized clinical trials (RCTs) may not generalize to individuals treated outside of these settings.

A recent meta-analysis summarizing the benefit of implementing EBPs over usual care showed that 58% of randomly selected youth receiving EBPs would have better outcomes than randomly selected youth receiving usual care (effect size 0.29) (7). Notably, none of the 52 RCTs included in this analysis were for eating disorders. The most efficacious EBP for adolescents with AN is family-based treatment (FBT), a manualized intervention that emphasizes the role of parental support in facilitating their child’s recovery from AN (8). An FBT approach consists of an average of 6–12 months of therapeutic intervention; the treatment includes three phases, whereby it is initially symptom focused, with parents providing meal support and prevention of compensatory behaviors with a primary goal of weight restoration. FBT differs considerably from other approaches such as individual therapy and inpatient management, in that parents are instrumentally involved in their child’s weight restoration, and resumption of appropriate eating and exercise behavior. Consistent evidence suggests that FBT is an efficacious therapy for this patient population, contingent upon medical stability for outpatient management (e.g., 9–11). However, research on provider attitudes towards the use of EBPs for eating disorders suggests that there are several barriers to using manualized treatments (12). Some argue that evidence supporting EBPs is flawed in important ways (13). In particular, it is thought that manualized treatment formats may be too rigid and not a “good fit” for most patients seen in community settings (14, 15). Therapists commonly endorse misconceptions and negative beliefs about FBT prior to training (16). Further, therapists who implement FBT often make significant modifications to treatment delivery (17), and in so doing, may unintentionally compromise the effectiveness of treatment.

To date, only one study of adolescents receiving FBT for AN compared the delivery of this treatment in a research trial (*n* = 32) versus usual care (*n* = 52) (18). These authors found that for adolescents with a lower initial percent median body mass index for age and height (%mBMI; 19), time to weight restoration was significantly faster in the research trial compared to usual care. However, for those with a higher baseline %mBMI, time to weight restoration was largely similar across care contexts. In this sample, psychiatric comorbidity was greater in usual care than in the RCT, suggesting that families of patients with elevated rates of comorbidity may be less likely to participate in research trials. In fact, Couturier and colleagues (12) have argued that a patient’s clinical complexity may discourage community clinicians’ use of FBT. Studies using highly controlled efficacy designs, where all providers involved in care are required to follow detailed treatment protocols (e.g., RCTs), might be expected to produce better outcomes compared with studies using effectiveness designs in which EBPs are evaluated under more usual practice conditions. Therefore, evaluation of FBT with a more representative sample in comparison to a research trial may be particularly informative.

The current study aimed to build on the work by Accurso and colleagues (18), who studied a private practice sample in the United States, where cost differs significantly between usual care (insurance or self-pay cost) and randomized trial care (treatment at no cost). Instead, the current study was conducted in a geographically restricted public health care setting in Australia, that is, the only specialist eating disorders service for this particular region with no direct treatment costs to the families receiving either treatment arm. Without cost barriers, it is posited that patients managed within such a public health care environment are perhaps more representative of the general population of adolescents with AN.

The primary goal of this study was to investigate time to weight restoration among patients who received FBT in the context of an RCT versus non-research specialty care delivered within an academic eating disorder service. While our study was largely intended to generate hypotheses, we anticipated a difference in time to weight restoration in favor of those participating in the RCT. The secondary goal was to test the potential moderating effect of baseline patient demographic and clinical characteristics on outcomes, given that the public health care setting of this study is likely to manage a more diverse sample when compared to the prior US-based study conducted in a private setting (18). Given the nascent evidence to date on nuanced differences in adolescent treatment outcome relative to moderators, secondary hypotheses remained exploratory.

## Methods

Participants were 110 adolescents who met *DSM-IV* criteria for AN, were medically stable, and were treated with conjoint FBT in an outpatient setting, either through an RCT (RCT Care: *n* = 54) or non-research specialty care (Non-Research Care: *n* = 56) from 2010 to 2016. Patients presented to the Royal Children’s Hospital, a tertiary public hospital in Melbourne, Australia. The Human Research Ethics Committee of the Royal Children’s Hospital approved this study, and participants provided consent or assent (RCT Care = written; Non-Research Care = written/waived) prior to participation. All therapists had specialized training and weekly supervision. Patients enrolled in the research study experienced differences in treatment delivery compared to Non-Research Care, including 1) sessions that were recorded, 2) random assignment to conjoint FBT, or a separated format of FBT, called parent-focused therapy (PFT), and 3) a requirement to be on a stable course of medication for a minimum of 8 weeks, or on no medication at all. The following reasons for exclusion were noted: 3 = medication; 5 = too young; 1 = too young and parents non-English-speaking; 3 = had FBT previously; 1 = had a medical condition. Reasons for declining participation in the study were as follows: 7 = no reason given; 5 = perceived burden (e.g., time, effort, stress); 2 = did not want to commit to protocol. A full description of the similarities and differences between RCT and Non-Research Care is detailed in **Table 1**.

**Table 1:** RCT Care vs. Non-research Specialty Care.

		RCT Care	Non-research Specialty Care		
**Setting**	*Location*	Highly specialized eating disorder program located in a tertiary care hospital
	*Medical/psychiatry care*	Provided by the team pediatrician and (if indicated) psychiatrist
	*Payment*	No-cost treatment
	*Wait list*	Brief (typically 2 weeks)	
	*Contact and assessments*	Frequent contact and assessments throughout treatment and follow-up with research staff
	*“Observation”*	Sessions audio taped (with consent)	Sessions not recorded
**Treatment**	*Implementation*	Fixed dose (18 sessions) of manualized FBT with high adherence required in implementation
	*Assignment*	Random assignment to FBT (versus parent-focused FBT)	Clinical recommendation to receive FBT
**Therapists**	*Degree*	Masters- and doctoral-level psychologists, or family therapy–trained social workers
**Training and Supervision**	*Training/supervision*	Structured training and supervision in FBT provided on a weekly basis with oversight of treatment adherence
**Patients**	*Referral route*	Clinical and personal referrals *via* a multi-disciplinary assessment clinic			
	*Diagnosis*	AN with %mBMI ≤90	
	*Age*	12–18 years	8–18 years
	*Medication* *Other characteristics*	Stable dose of medication >8 weeks (or no medication) No differences	No medication exclusion criteria

RCT, randomized controlled trial; FBT, family-based treatment; AN, anorexia nervosa; %mBMI, percent median body mass index; gray = similar; black = different.

### Research Trial Care (RCT)

The RCT sample (*n* = 54) participants were aged between 12 and 18 years, met *DSM-IV* criteria for AN (excluding amenorrhea) or Eating Disorder Not Otherwise Specified (AN type), were ≤ 90%mBMI at baseline, lived with at least one parent who was available to participate in treatment, and were evaluated for study participation between July 2010 and July 2014. Exclusion criteria were current psychotic disorder; drug or alcohol dependence; acute suicidality; physical condition influencing eating or weight (e.g., pregnancy); previous FBT; and psychotropic medication use <8 weeks. Data on the number of participants who were screened out for these reasons are reported in the main outcome paper (10).

### Non-Research Care

The Non-Research Care sample (*n* = 56) was mixed [13 not eligible for the RCT, 14 refused participation, and 29 not applicable (RCT recruitment completed)], drawn from children and adolescents aged 8–18 who were evaluated in the same outpatient eating disorders assessment clinic between August 2010 and November 2015, met *DSM-IV* criteria for AN or Eating Disorder Not Otherwise Specified (AN type), were ≤ 90%mBMI at baseline, and received FBT. The same cohort of eight clinicians who provided care in the RCT were available to provide care in this context. Therapists were doctoral- and masters-level psychologists or certified family therapists.

### Measures

Demographic and clinical characteristics were evaluated for all participants during an eating disorders assessment clinic visit. The same assessment battery was conducted at baseline, end-of-treatment, and at 6- and 12-month follow-up for both groups. For the purposes of the current study, we only note the measures of relevance to our primary research question (for the RCT protocol, see 20).

#### Weight and height

Patient weights were taken at baseline, week 4, week 12, end-of-treatment, and at 6- and 12-month follow-up; height was also regularly measured. Depending on context and availability, weights at weeks 4 and 12 were taken by 1) researcher (gown), 2) pediatrician/nurse (gown), or 3) therapist (lightly clothed).

#### Eating Disorder Examination

Eating Disorder Examination (EDE, 21). Diagnoses were determined by EDE interview, and its global score was used to determine baseline and subsequent eating disorder pathology. The EDE has demonstrated good reliability and validity (see 22, for review).

#### Child Depression Inventory

Child Depression Inventory (CDI; 23) is a 27-item self-report measure of cognitive, affective, and behavioral symptoms of depression in children and adolescents. Each item is scored on a three-point scale (0–2) according to symptom severity. The measure has demonstrated good reliability and validity (24).

### Statistical Analyses

To explore the extent to which RCT Care was comparable to Non-Research Care on baseline demographic and clinical characteristics, independent *t*-tests, and chi-square tests were used. Differences in treatment dose (i.e., total sessions, treatment duration) between types of care were also examined with *t*-tests. Survival analyses were used to compare the two samples on time to achieve 95%mBMI (25, 26). Individuals who were not weight restored by end-of-treatment (*n* = 73, 66.4%) were treated as “censored” observations, indicating that treatment response did not occur prior to termination of the measurement period. A Cox proportional hazard model was then fitted using a log logistic distribution. Chi-square tests were used to compare the proportion of patients who had achieved 95%mBMI at 6- and 12-month follow-up, according to treatment group.

The following baseline variables were initially examined in separate models (including main effects for the variable and type of care, and their interaction) as predictors of time to weight restoration: age, baseline %EBW, eating disorder pathology (i.e., EDE global score), duration of illness, psychiatric comorbidity, hospitalization prior to treatment, psychotropic medication use, depressive symptoms (i.e., CDI score), intact family status, and parent education. Dichotomous predictors were coded as −.5 and +.5, and continuous predictors were mean-centered (27). Main effects and interactions that significantly predicted time to 95% mBMI (*p* < .10) in their initial models were simultaneously entered into a final model. Factors specific to the therapist (e.g., personality, experience) were not determined in the data or included in analyses. IBM SPSS Statistics 25 was used for all analyses.

## Results

### Missing Data

Partial to fully complete data were available for all participants up to 6 months post-baseline. Weight data missing at week 4 was only evident for those in Non-Research Care [*n* = 4 (3.6%)]; at week 12, missing weight data were comparable for both groups [*n* = 6 (5.5%) RCT and *n* = 7 (6.4%) Non-Research Care]. Compared to those with complete data, patients with incomplete data were largely similar on eating disorder pathology, psychiatric comorbidity, hospitalization prior to treatment, psychotropic medication use, depression scores, intact family status, or parent education. However, those with missing weight data resulting from early treatment termination were older (15.6 vs. 14.7 years, *t* = −2.48 *p* = .02), had lower initial weights (79.3 vs. 84.3%mBMI, *t* = 4.19, *p* < .001), and has longer duration of illness (10.1 vs. 7.5 months, *t* = −2.44, *p* = .02). Weight data were available for all patients at their last treatment session (end-of-treatment), for 69 patients at 6-month follow-up (RCT: *n* = 50, 89%; Non-Research Care: *n* = 19, 34%) and for 84 patients at 12-month follow-up (RCT: *n* = 43, 79%; Non-Research Care: *n* = 41, 73%).

One participant had missing EDE data at baseline. While 36 (32.7%) were missing data on baseline parent education, these data were missing at random, based on a non-significant chi-square statistic (χ2 = .36, *p* = .84) for Little’s Missing Completely at Random (MCAR) analysis (28). The CDI was not scored if more than one item was missing, which resulted in 23 participants (21%) with missing baseline depressive symptom data. Evaluation of all CDI items in the full sample and per treatment group indicated these data were again missing at random, based on a non-significant chi-square statistic (χ2 = 288.1, *p* = .81) for Little’s MCAR analysis.

### Participant Characteristics

Baseline participant characteristics are shown in **Table 2**. The combined sample was primarily female (90.9%, *n* = 100) with a mean age of 15.3 years (*SD* = 1.9). Mean %mBMI was 81.0 (*SD* = 6.3), with an average duration of illness of 12.3 months (*SD* = 9.5). There were no significant differences between the two types of care in psychiatric comorbidity.

**Table 2:** Sample Characteristics at Baseline.

	RCT Care (*n* = 54)	Non-Research Care (*n* = 56)	*p*
Age (years), *M* (*SD*)	15.43 (1.33)	15.09 (2.25)	.34
Male, *n* (%)	6 (11.1%)	4 (7.1%)	.47
Australian born, *n* (%)	50 (92.6%)	54 (96.4%)	.30
Intact family, *n* (%)	35 (64.8%)	37 (66.1%)	.89
Parent education, *M* (*SD*)	9.56 (3.85)	8.89 (3.55)	.45
AN binge/purge subtype, *n* (%)	14 (25.9%)	13 (23.2%)	.74
Weight (%mBMI)	80.45 (5.41)	81.49 (7.08)	.75
Global EDE Score, *M* (*SD*)	2.12 (1.75)	1.76 (1.54)	.26
Duration of illness (months), *M* (*SD*)	11.07 (9.49)	13.50 (9.43)	.18
Co-morbidity, *n* (%)	19 (35.2%)	16 (28.6%)	.46
CDI Score, *M* (*SD*)	18.81 (10.87)	17.16 (9.23)	.45
Psychotropic medication, *n* (%)	6 (11.1%)	8 (14.3%)	.62
Hospitalization prior to FBT	21 (38.9%)	25 (44.6%)	.54

Parent education = combined mean of years of education of both parents; %mBMI = percent median body mass index for age and height; EDE, Eating Disorder Examination; CDI, Child Depression Inventory.

### Treatment Dose Across Type of Care

FBT was delivered in both arms over a course of 24 weeks (18 sessions); 15 patients (14%) (RCT = 7; Non-Research Care = 8) had extended treatment [2–7 extra sessions (one outlier had 14 sessions); and 5–14 extra weeks (one outlier had 35 weeks)]. RCT participants received a mean of 14.9 sessions [*SD* = 4.4; range: (4, 18)] over 19.5 weeks [*SD* = 6.7; range: (1.1, 26.1)]. Those in Non-Research Care received a mean of 14.4 sessions [*SD* = 4.9; range: (1, 18)] over 19.4 weeks [*SD* = 7.2; range: (0, 26.4)]. There were no significant between-group differences in treatment length or dose (all *p*s > .50).

### Time to Achieve Weight Restoration

Of the 110 participants, 37 (34%) were weight restored within 6 months of treatment (RCT: *n* = 19, 35%; Non-Research Care: *n* = 18, 32%). Across the full sample, the mean time to weight restoration was 5 months (*M* = 21.12 weeks, *SD* = 3.86). Of patients with weight data at 12-month follow-up (*n* = 84, 76%), 37 (44%) were weight restored (RCT: *n* = 19, 35%; Non-Research Care: *n* = 18, 32%); there was no significant between-group difference in proportion of those weight restored, according to treatment context (χ2 = 0.001, *p* = .98).

In all initial models, the main effects for treatment context, eating disorder pathology, illness duration, depression scores, parent education, intact family status, psychiatric comorbidity, psychiatric medication use, and hospitalization prior to outpatient treatment were non-significant (*p*s > .10). There were significant main effects for age (Wald chi-square = 5.19, df = 1, *p* = .02, OR = 3.62, 95% CI = 1.19–10.94), such that those who were younger were less likely to achieve weight restoration by 12-month follow-up. Those with a lower initial %mBMI were also significantly less likely to achieve weight restoration (Wald chi-square = 7.11, df = 1, *p* = .01, OR = 0.66, 95% CI = 0.48–0.89). There were no other significant main effects or interactions between variables of interest and treatment group (*p*s > .10).

**Figure 1:**
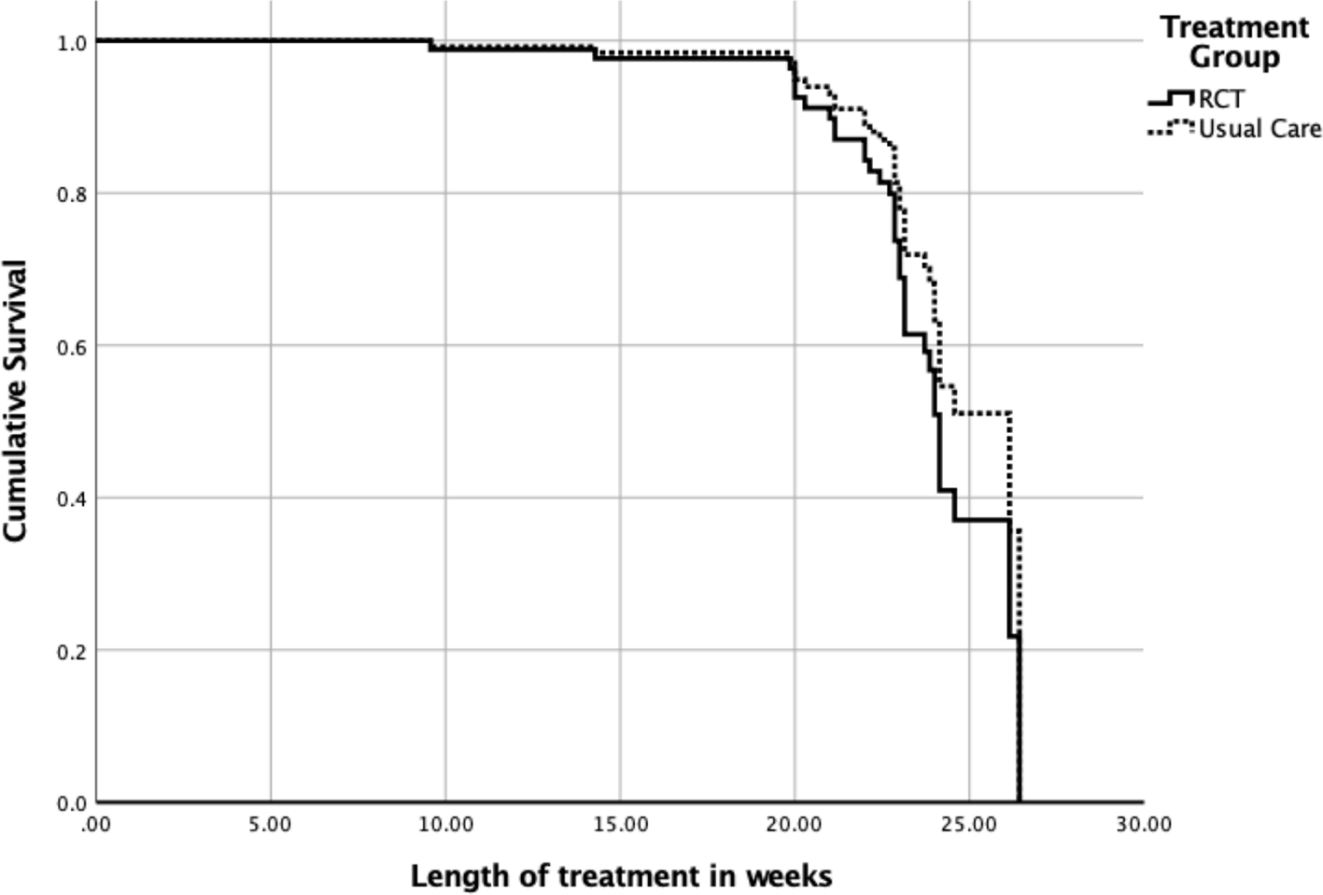
Time to weight restoration for randomized clinical trial (RCT) care vs. non-research specialty care.

The overall model included main effects for type of care, age, and %mBMI. There were 16 censored cases (RCT: *n* = 7, Non-Research Care: *n* = 9) before the earliest event in the stratum, which were dropped from final analyses. The overall model did not significantly predict time to weight restoration (overall chi-square = 6.13, df = 3, *p* = .11) (**Figure 1**). The main effect of %mBMI (B = .07, SE = .03, *p* = .03, OR = 1.07, 95% CI = 1.00–1.15) remained significant, such that weight restoration was achieved faster by those who had higher baseline %mBMI. The main effects of age and treatment context were not significant (*p*s > .10).

## Discussion

The current study compared weight outcomes in a sample of adolescents diagnosed with AN who received FBT *via* a research trial or non-research specialty care, both treatments provided within an academic specialist eating disorder service. Specifically, this study investigated time to weight restoration across these two groups, with a secondary aim to examine these trajectories relative to potential baseline moderators. At baseline, there were no between-group differences in any variables of interest (e.g., %mBMI, eating disorder pathology). However, across both treatment groups, those who were younger, or who had entered treatment with a higher weight, were more likely to achieve weight restoration within the 18 allotted sessions. Overall findings from survival analyses indicated that the rate of weight restoration did not appear to differ according to type of care (RCT vs. Non-Research Care) but that elevated baseline weight remained a significant predictor of achieving weight restoration more quickly within treatment. These results are not surprising given that patients who start treatment at a higher weight have less weight to gain in order to achieve 95%mBMI.

Baseline demographic and clinical variables suggest that the non-research specialty care sample of patients was quite similar to the sample who participated in the RCT. Further, when provided with the same treatment in the same clinic environment, the current study provides preliminary evidence that the more strictly implemented protocol of a trial did not appear to confer any significant advantage in achieving timely weight restoration, or differences in sustained weight gain at 6- or 12-month follow-up. Certainly, null findings can be difficult to draw conclusions from when sample sizes are modest or small, which is a potential limitation of the current study. Increasing the generalizability of research findings with comparable representativeness in study samples is of critical importance. This study supports the notion that FBT can be effective across a diverse patient population since it was conducted in a public health care setting, which admits all patients (geography as the only criterion for entry). However, the highly controlled implementation of FBT across settings, which typically only characterizes clinical trials, is difficult to replicate in true usual care settings. As a result, community providers may significantly modify EBP delivery (17), because of the constraints on clinical practice outside specialty centers (e.g., lack of training, supervision, support from a multidisciplinary team who further convey to families, the value of FBT), which may ultimately impact the likelihood of weight restoration at the end-of-treatment in non-specialty settings (29). This study does not inform how setting factors may influence implementation and outcomes in a usual, community-based setting.

Irrespective of RCT or non-research care, participants who entered treatment with a lower %mBMI were the least successful in achieving weight restoration by end-of-treatment. While this may not seem surprising in the context of adolescents needing to gain more weight, prior research found that time to achieve weight restoration was actually similar across %mBMIs, with the exception that for patients at a lower %mBMI, significantly fewer achieved weight restoration in the context of non-research specialty care compared to RCT care (18). Future work might further explore individual differences that contribute to improved treatment outcomes in order to maximize effectiveness of FBT for patients who begin treatment at different baseline weights.

The US-based (18) and current Australia-based study were conducted in academic settings with a history of undertaking clinical trials. In these highly specialized settings, research can examine whether differences in trial eligibility criteria (generally stricter within a trial) are associated with different rates of remission. As would be expected, clinicians who work in such settings are accustomed to delivering protocol-driven care, presumably comfortable with receiving supervision, and supportive of a research environment, so treatment will look similar whether or not a trial is underway. In the current study, approximately half of the Non-Research Care group was treated after the RCT; the site also had new clinicians starting in the midst of the combined time period, rendering any specific differences that could be attributable to clinician experience difficult to track. While this is a study limitation, it may also increase the overall generalizability of its findings to real-world settings. A critical difference between the US-based study (18) and our current study is that in a public health care setting, the ability to pay typically will not affect any family or provider decisions. In effect, we have demonstrated that within the constraints of the current sample size, which arguably is modest, and in consideration of the characteristics that were measured, there were no significant differences in eligibility or baseline characteristics (e.g., psychiatric comorbidities), and that treatment intensity appeared to be the same across the two treatment contexts. However, one important question that remains unanswered is how a “real-world” representative sample, receiving true usual care and drawn from a setting outside the context where research trials are conducted, will compare to research-based care delivered in an academic setting. Very little research has been done in this area. Preliminary efforts to address this question appear promising (12, 18), but there is still relatively little implementation science in the field of eating disorders. Implementation efforts are needed to further our understanding of treatment effectiveness in community-based usual care settings and the factors that impact implementation in order to maximize outcomes in these settings.

## Data Availability Statement

The datasets generated for this study are available on request to the corresponding authors.

## Ethics Statement

The studies involving human participants were reviewed and approved by The University of Melbourne, Melbourne, Australia. Written informed consent to participate in this study was provided by the participants’ legal guardian/next of kin.

## Author Contributions

Study design and conception: DG, EH, SS, and EA. Statistical analysis: EA and SG. Writing and review of manuscript: all authors.

## Funding

SG is supported by the National Institutes of Health (T32 grant MH0118261-33) (United States). This study was supported by a grant from the Baker Foundation (Australia). The Murdoch Children’s Research Institute is supported by the Victorian Government’s Operational Infrastructure Support Program.

## Conflict of Interest

DG receives royalties from Guilford Press and Routledge, and is co-director of the Training Institute for Child and Adolescent Eating Disorders, LLC.

The remaining authors declare that the research was conducted in the absence of any commercial or financial relationships that could be construed as a potential conflict of interest.
